# The Perinatal Adverse events and Special Trends in Cognitive Trajectory (PLASTICITY) - pre-protocol for a prospective longitudinal follow-up cohort study

**DOI:** 10.12688/f1000research.2-50.v1

**Published:** 2013-02-14

**Authors:** Laura Hokkanen, Jyrki Launes, Katarina Michelsson

**Affiliations:** 1 Faculty of Behavioural Sciences, Division of Cognitive and Neuropsychology, University of Helsinki, Helsinki, Finland; 2 Faculty of Medicine, Department of Neurology, University of Helsinki, Helsinki, 00029, Finland; 3 Children's Hospital, University of Helsinki, Helsinki, 00029, Finland

## Abstract

Prospective follow-up studies on long term effects of pre- and perinatal adverse conditions in adulthood are rare. We will continue to follow the prospective cohort of initially 1196 subjects with predefined at-delivery risk factors out of 22,359 consecutive deliveries during 1971-74 at a single maternity hospital. The risk cohort and 93 controls have been followed up with a comprehensive clinical program at 5, 9, and 16 years of age and by questionnaire at the age of 30 years. Major medical events known to affect the development and growth of the brain, or cognitive functions and personality have been documented. Here we present a pre-protocol for the project, which we will call PLASTICITY, whose aim is to follow consenting subjects and controls into mid-adulthood and beyond, and to explore how the neonatal risk factors modulate neurodevelopmental and neurodegenerative processes such as learning disabilities, ADHD, aging, early onset mild cognitive impairment and even dementia. Our first focus is on the neurological and cognitive outcomes at age 40 years, using detailed neurological, neuropsychological, neuroimaging, genetic, blood chemistry and registry based methods. Results will be expected to offer information on the risk of neurological, psychiatric, metabolic and other medical consequences as well as the need for health and social services at the brink of middle age, when new degenerative phenomena are known to emerge. The evaluation at age 40 years will serve as a baseline for later aging studies. We welcome all comments and suggestions, which we will apply in finalizing details and inviting collaboration.

## Introduction

We present here pre-protocol of the "PerinataL Adverse events and Special Trends In Cognitive TrajectorY" (PLASTICITY) study. The study aims to test the hypothesis that perinatal adverse events exert an unexpectedly deleterious effect on the brain at middle and older age. This is a prospective longitudinal at-risk cohort study of a 1971–1974 birth cohort that has been prospectively followed to adulthood ^1^. Several papers, theses, and book chapters about the findings in children up to the age of 16 years have been published based on this material ^2– 11^. Therefore, we know the individuals that are in different outcome groups and etiologies. All consenting subjects are aimed to be studied now in their 40s and throughout their remaining lives at 10 or 5 year intervals.

This paper describes our current plans on how this cohort is to be followed-up. An enormous amount of work has been done in the first decades of this follow-up study of individuals from at-risk deliveries. We feel that the unique opportunity of completing the follow-up of such a cohort from birth to death must not be missed. However, we acknowledge that without collaboration, we cannot achieve all that could and should be done. Therefore, we welcome all comments and suggestions, which we will apply in finalizing the study details and inviting collaboration. While a general schedule is outlined here, the exact timetable will depend on methodological, administrative, and financial decisions.

## Background

Antenatal and perinatal risks of infant death or disability are well known. These include intrauterine growth restriction, ischemia/hypoxia/asphyxia ^12^ due to many reasons, jaundice ^13, 14^, infection, drugs and maternal disorders ^15, 16^. There is a wealth of general literature about the diagnosis, etiology, treatment, social consequences and individual outcomes, and the range of conditions reaches from mild defects to cerebral palsy, serious cognitive deficits and death ^2, 12, 17– 30^. The incidence of death or considerable disability is 0.2 to 5 out of 1000 live births in hypoxic-ischemic encephalopathy ^31, 32^, intrauterine growth restriction ^23, 33– 36^, or jaundice ^21^. The outcome has been improving steadily, e.g. according to the Official Statistics of Finland ( http://tilastokeskus.fi/til/kuol/tau.html), infant mortality has decreased over 70% from 12.7 by 1000 births in 1971 to 2.7 by 1000 births in 2007.

The timing, type, and severity of the long term consequences of antenatal and perinatal complications have mostly been studied in relatively short follow-up studies ^3, 8, 9, 17, 19, 23, 28, 32, 33, 36– 41^. Most of them cover the period up to early school age, with possibly some overemphasis on cerebral palsy due to its juridical importance in many cases ^42, 37^. In studies focusing on cognitive, neuropsychiatric and social performance, the longest follow-up periods currently extend into young adulthood ^33, 43, 44^. Long term studies report diminished IQ and/or scholarly achievement but these are studies that are either retrospective or rely on data from secondary sources such as tests for conscripts ^13, 45, 46^. Recent results of a retrospective longitudinal study using more specific cognitive tests indicate impaired psychomotor speed, learning and executive functioning in young adults with very low birth weight ^43, 47^. Many very interesting and ambitious longitudinal prospective cohort studies have been started recently (see for instance www.birthcohorts.net) but the subjects in the actively followed-up prospective birth risk cohorts we are aware of ^48– 53^ will not reach an age when aging-related changes have a serious effect for decades. To the best of our knowledge, no results of prospective follow-up beyond young adulthood exist to date.

The types and extent of structural abnormalities caused by intrauterine growth restriction and/or asphyxia are well known in seriously disabled children. It has been possible to investigate the more subtle changes in brain structures *in vivo* only relatively recently. So far there are relatively few MRI studies ^25, 41, 54– 60^ of children who were exposed to adverse conditions *in utero* or perinatally. However, a wide variety of subtle abnormalities resembling those caused by many diseases in adult life, especially in the brain white matter, have been discovered. The current knowledge about either normal or pathological aging emphasize the role of white matter changes both in degenerative and vascular pathology, e.g. white matter lesions are independent risks in both ischemic stroke, vascular dementia, and also degenerative dementias. The time course of these changes is as yet very poorly known.

The incidence of Attention Deficit Hyperactivity Disorder (ADHD) is known to be higher among the subjects with pre- and perinatal risks ^61, 62^. In a recent study, low birth weight, preterm birth, and low Apgar scores were reported to increase the risk of ADHD up to 5-fold ^63^. Other syndromes of childhood developmental disabilities include reading disorder/dyslexia, non-verbal learning disorder, dyscalculia, and disorders of motor coordination; entities that often overlap and coincide. The etiology of many of these developmental disorders involves the interaction of multiple risk and protective factors, both genetic and environmental ^64– 66^. Follow-up studies suggest that in a large number of children ADHD persists in adulthood, but the range in the reported frequencies is wide, 5–66% ^67^. The symptoms may change during lifespan, hyperactivity becoming less common ^68, 69^ in adulthood. Prospective follow-up studies spanning into the ages of 30–40 are extremely few and none so far reach beyond 40 ^70, 71^.

As people reach adulthood and beyond, they become susceptible to the neuronal effects of ageing. Based on both large population based cohorts and clinical follow-up studies, most cognitive scores appear to decline from the age of 45 onwards, with faster decline in older people ^72, 73^. Along with the normal ageing process, pathological processes also start evolving, and the distinction is clinically not easy to make. Mild Cognitive Impairment (MCI) refers to a preclinical stage that converts to dementia as the disease progresses ^74, 75^. On a clinical level MCI is a useful concept but neuropathologically it is complex and inadequately understood ^76^. The classical markers of Alzheimer's disease (AD) neuropathology start to appear in 40-year-olds ^77^ with a clear correlation to cognitive functioning ^78^. Terms describing preclinical states of AD (including both "asymptomatic at-risk state for AD" and "presymptomatic AD") refer to the long asymptomatic stage between the earliest pathogenic events/brain lesions of AD and the first appearance of specific cognitive changes ^79, 80^. There is evidence that mid-life levels of cardiovascular risk factors (such as elevated blood pressure, cholesterol and smoking) increase the risk for diseases affecting cognition that emerge 20 years or more after the risk factor is measured ^81^. The role of the vulnerability factors that have been present from the neonatal period in this progression is not known.

Approximately 8% of all dementia cases are in working age (from 30 to 65 years) ^82^ with the estimated prevalence among the 45–64 year age group being 98.1 per 100,000 ^83^. In both late and early onset dementias AD is the most common cause but frontotemporal degeneration and hereditary forms of dementia are more prevalent in the early onset group ^82, 84^. The significance of rarer and ‘disregarded’ pathologies to late-onset dementias has recently been explored in an epidemiological study ^85^ but the neuropathological mechanisms of early onset dementias are not fully understood. The significance of perinatal and early childhood events in relation to MCI and dementia are unknown, although asphyxia and preterm birth are listed as risk factors. Based on statistics only, approximately 300 subjects in our birth cohort will develop dementia ^86, 87^.

## Rationale

Our aim is to identify and study the type and severity of changes that can be revealed by neurological, cognitive, psychiatric, neuroimaging, and neurophysiological techniques as well as metabolic and genetic analyses in a cohort of subject with predefined neonatal adverse events by means of a careful and lifelong follow-up. The results will be compared to those of peers born healthy to test our hypothesis that birth complications may cause undue damage to the central nervous system at a later age.

The rationale is based on the hypothesis of lowered cognitive reserves following perinatal adverse events which would lead to susceptibility for later damage. The concept of brain reserve is based on the protective potential of anatomical features such as brain size, neuronal density and synaptic connectivity ^88^ and it can be seen as passive, postulating that there is a fixed threshold below which functional impairment will occur ^88, 89^. In contrast, behavioral brain reserve, or cognitive reserve, is an active construct, suggesting that the brain actively attempts to cope with brain damage by using pre-existing cognitive processes or by enlisting compensatory processes ^89, 90^. Cognitive reserve is not fixed at any point in life; instead, complex interactions exist between genetics, environmental influences, and the ability to actively compensate for the effects of pathology. Further, it has been suggested that the possible neural implementation of cognitive reserve be subdivided into two components that can also be studied using neurophysiological methods: neural reserve, which refers to individual differences in cognitive processing paradigms and neural networks that are in use in the brain, and neural compensation, which refers to alterations in cognitive processing networks that may take place in order to cope with brain pathology ^89^. The risk cohort serves as a model for studying both the expression of cognitive reserve on later neurological conditions, and for evaluating the neural reserve and compensatory mechanisms.

**Figure 1.**
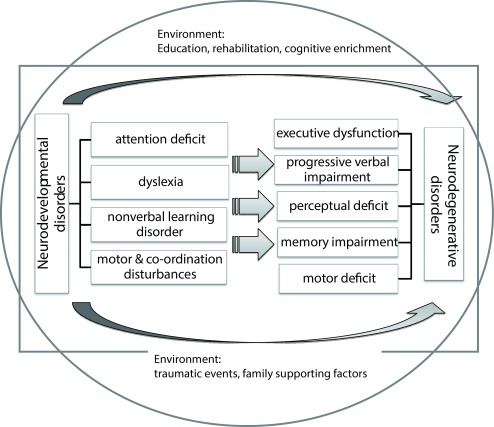
Schematic presentation of the study rationale. Phenotypes of the developmental disorders are suggested to modify the phenotypes of age-related degeneration while individual, genetic and environmental factors interact. Perinatal adverse events may potentially pose a lifelong burden by decreasing the cognitive reserves of the individual. Genetic and epigenetic variables set the framework for both early development and the ageing process. Environmental factors may initiate both a positive and a negative cycle while education and rehabilitation can enhance neural reserves and neural compensation.

Cognitive reserve has typically been estimated by means of autobiographical data such as socioeconomic status, occupational complexity, educational level, and mentally stimulating leisure activity, in addition to specific measures of IQ. A considerable number of cohort studies have shown the protective effects of these variables in incident dementia (see reviews ^91– 93^). Both exercise and cognitive stimulation regulate factors that may increase neuronal plasticity, and there is evidence to suggest that environmental enrichment might act directly to prevent or slow neurodegenerative disorders and permit normal cognitive functioning even in the presence of brain pathology ^94^. Similar modulation probably exists in neurodevelopmental disorders, and these factors will be included in the study paradigm. Figure 1 illustrates the rationale of the project PLASTICITY.

## Aims and objectives

In the present project, several parallel approaches will be applied.

1) **Etiology based approach**: Study the neurological, neuropsychological and neuropsychiatric outcome in adulthood in relation to antenatal- and perinatal events that have been established in follow-up of our subjects (e.g. asphyxia, low birth weight, hyperbilirubinemia, maternal diabetes).

2) **Syndrome based approach**: Assess long term effects of commonly recognized neurodevelopmental deficits (such as dyslexia or ADHD), to explore the associated neuropsychological, neurological and neuropsychiatric symptoms in adulthood, and deepen and broaden existing knowledge of such symptoms.

3) **MCI** at the age of 40: Recognize the potentially elevated risk for cognitive decline in the group of adults with a history of pre- and perinatal risk factors; to study the prevalence of MCI as well as early onset dementia in the working age. Special emphasis will be given to individuals, who have been diagnosed with asphyxia and/or have white matter findings in MRI.

4) **Cognitive follow-up**: Assess how the different developmental deficits and factors affect and transform into different varieties of MCI/dementia during later age (repeated evaluation cycles at the age of 50, 60, 65, 70, 75 etc. years).

5) **Radiological follow-up**: Define neuroradiological findings in adults with pre- and perinatal risk factors; to clarify the structural lesions in adulthood and to use this information as a baseline for the MCI/dementia follow-up.

6) **Genetic analyses**: Analyze the known risk factors of neurological and psychiatric traits (e.g. APOE, DISC1, COMT, ROBO1 to name a few) to be used as covariants in the assessment of disease risk in the whole cohort; genome-wide association study (GWAS) of clearly defined traits, including dyslexia, specific forms of cognitive decline, MRI parameters (leukoaraiosis, regional atrophy etc.). As medical genetics is at the moment the fastest developing of all modalities, this approach will have to be continuously reevaluated.

7) **Metabolic and endocrine effects**: Measure metabolic and endocrine functions to be used as important cofactors in statistical analysis. Previous data suggests that low birth weight, preterm delivery and asphyxia may cause various metabolic effects e.g. diabetes and other endocrine abnormalities. On the other hand, these are known to influence the normal as well as the pathological aging process independently.

8) **Protective brain plasticity**: Acknowledge the capacity of the brain for plasticity and neural compensation. The length and type of education, amount of special education and rehabilitation, and participation in cognitively stimulating hobbies, exercise and other activities indicating cognitive enrichment will be assessed. We also aim to test the cognitive reserve hypothesis directly by studying the efficiency of neural networks with evoked potential and functional imaging techniques in a subsample of subjects.

9) **Adjustment, lifestyle, psychiatric comorbidities and quality of life**: Recognize the potential for psychological and psychiatric concerns. Data from questionnaires will be combined with registry data from national social and health registers as well as with the relevant clinical data.

## Design and subjects

### The 1971–74 birth risk cohort

The basis of this study is the 1971–1974 birth cohort from a Helsinki metropolitan area maternity hospital (Kätilöopisto hospital) that has been prospectively studied up to adulthood ^1^. There were 22,359 consecutive births which accounted for approximately 10% of all births in Finland during that time. At birth, 1196 (5.4%) presented with at least one predefined risk, see Table 1.

**Table 1.** Number of risk factors at inclusion into the study and at 5 years. First column (at inclusion) lists the total numbers of cases in each risk category; a case may appear in several categories. Second column (at 5 yrs) lists the numbers of cases with one risk factor only, and the number of cases with multiple risk factors is given separately.

	At inclusion (1196 cases)	Examined at 5 yrs (845 cases)
Alive at evaluation	1196	1038
Cases with risk factor		
Birth weight 2000 g or less ^1^	317	119
External ventilation	161	21
Apgar <7 at 5 or 15 min	372	138
Neurological symptoms: marked hypotonia, apathy, hyperexcitability, rigidity, convulsions, apnoeic spells	195	55
Hyperbilirubinemia: at least two serum bilirubin values of 340 µmol/l (20 mg/100ml) or more, or blood exchange transfusion	368	257
Hypoglycemia: at least two blood glucose values of 1.67 mmol/l (30 mg/100ml) or less for full term babies and 1.21 mmol/l (22 mg/100ml) or less for preterm babies (less than 37 gestational weeks)	104	38
Diabetic mother, including White A class	93	47
Septic infection, bacteriologically verified	36	7
Cases multiple risk factors		163
Ischemia/asphyxia ^2^	377	255

^1^ Note that 2000 g was considered a low birth weight, but 1500 g is used in some analyses.

^2^ Number of cases where significant ischemia/hypoxia was diagnosed after inclusion in the study.

Of the 1196 infants, 158 died before the age of 5. Additionally, 25 were severely disabled and were excluded from further analyses. At the first follow-up at 5 years, 67 could not be traced and 101 were unable to participate, therefore 845 children (462 boys, 383 girls) were re-assessed (at least 83% of those alive and not severely disabled) ^3^. Asphyxia is defined as any brain damage caused by direct ischemia or ischemia induced inflammatory response. These are the patients who were included in the study according to the criteria above and who simultaneously had objective findings indicating probable inadequate brain blood supply.

At the age of 9 years, 748 children were re-assessed and at the age of 16 years, a survey using a mailed questionnaire about neurodevelopmental symptoms was conducted. There were 521 responders. Of these, 142 children were clinically examined (mainly those with observed deficits at the age of 5 or at 9 years). At the age of 30 years, a survey was again conducted and 509 subjects responded.

Details of clinical examination and other assessments are given in Table 2.

**Table 2.** Tests and measures used at different phases of the study thus far. See footnote for the complete names of the tests. Group sizes (n), RG = risk cohort group, CG = control group.

	At birth RG n = 1196	5 years RG n = 845 CG n = 58	9 years RG n = 748 CG n = 165	16 years RG n = 521 (142 ^1^) CG n = 102 (25 ^1^)	30 years RG n = 509 CG n = 93
Maternal perinatal data	smoking diabetes medication blood pressure				
Medical	laboratory parameters		psychosomatic	use of alcohol use of drugs	use of alcohol use of drugs smoking accidents mood diseases medication
Neurology	observational scoring	Bax/structured hearing visus handedness	modif./structured audiometry visus handedness	"Soft signs"/structured	
Family history	illnesses	illnesses handedness	illnesses handedness		
Social environment	parents’ work situation	parents’ work situation Security risk scale Social risk scale	parents’ work situation Security risk scale Social risk scale	parents’ work situation	working history
Motor	observational scoring	Berges-Lezine	TOMI Stott Berges-Lezine Gubbay test	TOMI Stott tapping Luria praxis	
Speech/logopedic		articulation name writing	articulation writing	articulation writing	
Cognitive		ITPA Dubowitz	ITPA WISC	WAIS WMS Benton	
Visual perception		Frostig Goodenough	Goodenough	Clock & map	
Behavioral observation	observational scoring	observational scoring	observational scoring		
Behavior & personality rating			teacher rating	parent rating CBCL YRS	Barkley Scales
School achievement			grades special education needs	grades special education needs	grades education level

ITPA = Illinois Test of Psycholinguistic Abilities, TOMI = Test of Motor Impairment, WISC = Wechsler Intelligence Scale for Children, WAIS = Wechsler Adult Intelligence Scale, WMS = Wechsler Memory Scale, CBCL = Child Behavior Checklist, YSL = The Youth Self Report.

^1^ attended the clinical examination.

### Healthy control subjects

A control group of 58 children born in uncomplicated deliveries at the same maternity hospital has been followed from the age of 5, and 111 additional children from the age of 9 years of age. Out of the total 169 control cases, 93 returned the questionnaire at the age of 30 years. The control subjects were born at the same maternity hospital during the study period and mostly attended the same primary schools. Hospital records have been reviewed to confirm absence of any perinatal risk factors.

### Enrollment and attrition

Presently, the youngest subjects in the cohort are 39 and the oldest are 42 years of age. A full clinical follow-up will be performed during 2013–14, before the oldest subjects turn 43.

The goal is to enroll as many as possible of the original risk cohort, even if they had not been able to participate in some of the earlier follow-up cycles. All surviving cases in the cohort will be contacted with the exception of the severely disabled and those who did not express consent in the survey at 30. We estimate to be able to recruit at least 60% of the entire surviving risk cohort (n = 1038) for the clinical assessment, based on the responses of the 509/845 cases who have already consented to follow-up.

The control group included 93 cases at the time of the previous survey and we hope to be able to recruit at least 60. This longitudinal control group is important because they have shared the early life experiences, school and social circumstances with the risk cohort.

For the purposes of future follow-ups, a new control group in addition to the longitudinal controls is needed due to attrition and accumulating differences in social and economic surroundings. The new control group will therefore be recruited from the spouses of the risk cases because they share the current social environment and living conditions.

Attrition is a problem in all longitudinal studies. In Finland, as well as in other Nordic countries, requests to participate in research projects are usually met with a positive attitude and inclusion and dropout rates are known to be quite acceptable ^95^. Also, people to be contacted for recruitment are easily found through national registries. Still, specific measures to encourage subjects’ motivation to continue in the project are needed. Strategies to increase retention will be actively sought and good examples from ongoing projects will be followed (such as ^49^). These include, for instance, lowering the threshold to participate (compensation for travel costs, compensation for lost time, reminders to return questionnaires and flexible schedules for visits), inducing gains and benefits from participating (individual feedback on the medical results), as well as generating a general sense of availability and openness (websites, dissemination of the general results, contact opportunities via phone and email). Subjects will, however, not be paid for participation.

## Methods and outcomes in childhood

### Previously collected data

The database contains detailed data about the family’s social and economic status, maternal risk factors, family genetic traits, medical data about delivery and delivery complications, child’s growth and medical follow up at 5, 9 and 16 years. Additional Child Health Centre information was collected at the ages of 6 months, 12 months, 18 months, 24 months and 4 years. Surveys have included both parents’ and teachers’ questionnaires as well as self-reports, such as the Child Behavior Check List (CBCL) ^96^ and Youth Self Report (YSR) ^97^. A structured neurological assessment of the children was carried out using the Neurodevelopmental Screen developed by Michelsson *et al.* ^4^, a modification of the test of Bax and Whitmore ^98^ that also includes items from the Berges-Lezine imitation of gestures as well as Gubbay ^99^ test. Other standardized tests and measures used over the course of the follow-up include the Stott Test of Motor Impairment ^100^, Neurological "Soft signs" in Adolescence ^101^ Dubowitz developmental screening test ^102^, The Illinois Test of Psycholinguistic Abilities ^103^ (ITPA Finnish version ^104^), Goodenough Draw-a-person test ^105^, Frostig Developmental Test of Visual Perception ^106^, subtests from the Wechsler Intelligence Scale for Children ^107^ (WISC Finnish version ^108^) and subtests from the Wechsler Adult Intelligence Scale ^109^ (WAIS Finnish version ^110^). At the age of 16 part of the cohort was also assessed with more detailed neuropsychological instruments including the Benton Visual Memory Test ^111^ and subtests from the Wechsler Memory Scale ^112^ (WMS Finnish version ^113^). Table 2 shows the various measures categorized by function as well as the age of the subject when tested.

Adult outcome was surveyed with a questionnaire about education and work history, medical and social wellbeing as well as cognitive and psychiatric symptoms at 30 years of age. The questionnaire also included the Barkley Current Symptoms Scale as well as the Childhood Symptoms Scale ^11^.

### Medical and neurodevelopmental outcomes by diagnoses

The initial purpose of the study was to follow up newborn children with perinatal risk factors into adolescence to estimate the impact of low birth weight, bilirubin etc. on later development. Outcome measures were divided into 1) Medical, e.g. mortality, major disabilities, anomalies, learning disabilities; 2) Psychometric, e.g. development of the linguistic, cognitive and motor skill as assessed by standardized tests; 3) Achievement based, e.g. school performance, education, and work status; and 4) behavioral and social parameters.

The first results of the prospective follow-up research project were described in 1978 ^1^. Perinatal mortality in the risk cohort was 5.35% and it accounted for 83% of all perinatal deaths in that hospital during the study period 1971–74. Except for hyperbilirubinemia, which was less frequent in 1974, there was no marked change in the risk profile from 1971 to 1974 ^1^, indicating that no major breakthrough in treatment success occurred during that time.

The neurodevelopmental screening test performed at 5 years revealed the highest impairment scores in children with neonatal neurological disorders – which most likely had ischemic etiology – and lowest in those with neonatal septic infections ^3^. After the 5 year assessment, 42% of the children were referred for further assessment and/or rehabilitation measures such as special kindergartens, speech therapy, psychologist assessment or neuropediatric rehabilitation in a specialized centre ^115^.

Of the children with a birthweight of 1500 g or less, 50% died during the first 6 months ^6^. Of those surviving, 12.3% had severe motor, mental or sensory disabilities and even those without were found to have impaired motor function, speech defects and impaired school achievement more often than the controls ^6^. The children with a birth weight of 2000 g or less showed a similar but milder picture: mortality during the first 6 months was 28%, severe disabilities were present in 9%, and those without severe disabilities were found to show more impairment in neurodevelopmental screening examinations and in psychological and articulatory tests at the age of 5 years compared to controls. According to the teachers’ assessment at the age of 9 years, they were more often in need of special education compared to the controls ^5^. Also the children with neonatal hyperbilirubinemia managed less well in neurological and psychological tests at the age of 5 years. They had poorer school grades and more often attended special classes for somatically or mentally disabled at the age of 9 years, but their results were still often better than in the rest of the risk group ^11^. The children born during 1972–73 were analyzed for minor and major congenital anomalies. Those with anomalies were found to perform worse in cognitive and motor tests at the age of 9 compared to the other children in the risk group ^10^. The number of anomalies in the risk group was comparable with the control group but there were more small for gestational age children in the anomaly group than in the non-anomaly group ^10^.

### Minimal brain dysfunction/ADHD

A specific feature in a longitudinal study such as this one is the change in the diagnostic criteria over the years. The initial aim of the prospective study design was to trace children who showed signs of minimal brain dysfunction (MBD), a term which at the time incorporated both behavioral and learning disturbances and various combinations of deficiencies in perception, language, memory, attention, impulse and motor control ^116, 117^. The Diagnostic and Statistical Manual of Mental Disorders (DSM)-II in 1968 included a concept of ‘‘hyperkinetic reaction of childhood’’ and in the following version, DMS-III in 1980, this was substituted with attention-deficit with or without hyperactivity. MBD as a diagnostic or even descriptive term was mostly discarded thereafter.

The prevalence of hyperactivity at the age of 9 was reported to be higher in the study cohort than in the controls ^10^. When the current DSM-IV criteria were retrospectively applied to the childhood data, it was estimated that the cohort includes 122 cases with ADHD (attention deficit hyperactivity disorder) and their long term outcome will be published separately.

The current DSM-IV recognizes three ADHD subtypes, predominantly inattentive, predominantly hyperactive-impulsive, and a combined subtype. A new version, DSM-V is expected to be published in 2013, and the diagnostic criteria may again change. Old diagnostic groups will be retained in the database but classification of the subjects is constantly updated based on new emerging criteria.

## Methods and outcomes in adulthood

### Planned measurements in midlife

With the exception of the subgroup with suspected disorders who were contacted at 16, the majority of the cohort subjects have not been clinically evaluated after the age of 9. None have undergone MRI scans. It is therefore essential to thoroughly assess the whole group in order to have exact data on the adult outcome.

In the next cycle of assessment at 40 years of age, we are interested in the long term outcome of the developmental disorders dyslexia and ADHD in particular. We are also interested in whether the perinatal risk factors are associated with acquired neurological disorders. Particularly we want to explore the vascular system of the brain, focusing on the subjects with perinatal asphyxia. Later in midlife, at ages 50 to 60 years, the focus will gradually shift towards neurodegenerative disorders, and the study outline will later be updated accordingly.

The study outcomes, which are considered relevant for the risk group in middle age at 40 years, and also to 50 and 60 years assessment cycles, are outlined in Table 3.

**Table 3.** Outcomes to be investigated in the risk cohort in midlife, and methods to assess them.

Outcome	Foreseeable methods for analysis
ADHD and other learning disabilities	• Neuropsychological tests • Registry data harvesting • Psychiatric assessment • Assessment by significant others • Genetic testing
Acquired diseases	• Neurological examination • Screening tests e.g. Mini Mental State Examination (MMSE) • MRI • Blood tests targeted for e.g. diabetes and endocrine dysfunction • All other relevant clinical tests for any condition requiring medical attention • Registry data harvesting • Genetic testing
Normal aging	• Balance evaluation using body sway measurements • Gait and posture observation • Dexterity tests • Neurological "soft signs" • Screening tests e.g. MMSE
Mild Cognitive Impairment (MCI)	• Neuropsychological testing • Specific memory and attention tests • MRI • Functional MRI (fMRI) • Neuronal blood/serum markers • Genetic testing • Cognitive evoked responses with MRI and/or fMRI
Early onset dementia	• Neuropsychological testing • MRI • fMRI • Genetic testing • Neuronal blood/serum markers • Screening tests e.g. MMSE • Registry data harvesting • Risk assessment
Cognitive reserve and neural compensation	• Registry data harvesting • Interviews at visits • Inquiries and self-assessments • Assessment by significant others • Cognitive evoked responses with MRI and/or fMRI

**Neurological and medical examination** will include e.g. structured neurological history and status, hearing, vision, Mini Mental State Examination (MMSE), cardiovascular status, blood pressure, metabolic indices, measure of head circumference, handedness, dexterity, and body sway.

**Psychiatric disorders** will be screened by SCID-I ^118^ and SCID-II interviews ^119^. Specific tools for ADHD will include the Conners’ Adult ADHD Diagnostic Interview for DSM-IV (CAADID) ^120^ or the Diagnostic Interview for Adult ADHD (DIVA) ^121^.

**Neuropsychological assessment** at the age of 40 will include a battery of tests for basic visuospatial and visuoconstructive skills, tests for motor praxis and dexterity; tests for phonological processing, naming (Boston naming ^122^, Rapid alternating stimulus naming ^123^), reading, and arithmetic; executive function measurements (Trails Making Test, Word Fluency, Tower test and Color Word test, either from Delis–Kaplan Executive Function System ^124^ or as stand-alone tests), computerized tests for reaction time and attention (such as Continuous Performance Test ^125^ and Attention Network Test ^126^), tests for memory (such as Rey Auditory Verbal Learning ^127^ and Benton ^111^); as well as subtests of the Wechsler tests (WAIS-IV ^128^ and WMS-III ^129^). There will also be questionnaires for subjective symptoms and mood.

The same or slightly modified battery of tests will later be used in repeated testing. A particular challenge in planning the battery to use in longitudinal studies is the availability of the tests in years to come. The traditional pen and paper tests will be around but newer computerized methods present the risk of being more short-lived in the ever-evolving technology.

**Laboratory assessment and genetic analyses** cannot all be anticipated at the moment. Blood samples will be taken and stored until genetic analyses (e.g. for *APOE*, *DISC1*, *COMT*, and *ROBO1*) can be performed as a batch process. These are open to discussion and collaboration is actively sought.

**Neuroradiological imaging** including MRI (T1 and T2 weighted and FLAIR T2 imaging, diffusion imaging with diffusion tensor imaging, angiography, and volume measurements of the hippocampi, corpus callosum, relevant nuclei and other relevant structures that have to be defined ad hoc). Ideally, the MMSE would be scheduled the same day or at least within one week of the neuroradiological imaging session. Brain activity will be measured in selected cases using functional MRI (fMRI), recordings of event related potentials (ERP), electroencephalography (EEG) and magnetoencephalography (MEG).

**Registry inquiry** In addition to the clinical assessment, health register data will be gathered from the Finnish Social Insurance Institution (Kela) concerning disability benefits, health security, rehabilitation, and unemployment benefits. From the Finnish National Institute for Mental Health and Welfare (THL) register data will be applied concerning diagnoses from the National Hospital Discharge Register.

### Measurements at age 65 years and older

Longitudinal data will be collected as long as the subjects are willing to participate. We know from Finnish statistics that the estimated life expectancy for someone having reached 30 years of age in 2003 is 46 years for males and 52 for females ^130^. This would mean that the men in this cohort should live up to 76 and the women up to 82 years of age. The project outline will later be updated to include the studies after 65 years of age.

### Research group and collaborators

**Laura Hokkanen**, PhD, professor of clinical neuropsychology, University of Helsinki, is the Principal investigator. Other members of the research group include **Jyrki Launes**, MD, PhD, specialist in neurology, University of Helsinki, **Marja Laasonen**, PhD, Helsinki University Central Hospital, Department of Phoniatrics, **Anna-Mari Tuulio-Henriksson**, PhD, Kela – The Social Insurance Institution of Finland and **Maarit Virta**, PhD, University of Helsinki, Institute of Behavioral Sciences. Master’s and doctoral level students will be recruited in the project.

Collaborators at this point include **Kimmo Alho,** Professor of psychology, University of Helsinki, Helsinki Collegium for Advanced Studies and Institute of Behavioural Sciences, **Taina Autti**, MD, PhD, Professor of radiology, University of Helsinki, **Oili Salonen**, MD, PhD, Helsinki University Central Hospital, Department of Radiology, **Sami Leppämäki**, MD, PhD, Helsinki University Central Hospital, Department of Psychiatry, and **Pentti Tienari**, MD, PhD, Helsinki University Central Hospital, Department of Neurology and Biomedicum, University of Helsinki, Molecular Neurology Research Program.

National as well as international collaboration is invited. Please send comments and suggestions to Dr Launes at plasticity@live.fi.

## Data analysis and statistical plan

The original database created in 1971 was non-electronic (punched cards). It was later keyed in and analyzed using the BMDP (Statistical Software, Inc 1983). Currently the database is on PASW Statistics, Release Version 18.0.0 (SPSS, Inc.) and Microsoft Excel 2010 and can thus be converted and transported easily. The integrity of the data has been checked during conversions and will undergo continuous error checking both electronically and manually.

Interestingly, the structure of the database reflects the change in the information processing techniques over the past 40 years. Initially, due to the dichotomous nature of the punched card processing, the variables concerning the neonatal period and the first 5 years are mainly stored in a categorical/discrete format. This limits the statistical approaches as non-parametric statistics must be used. This, however, in no way prevents the use of early perinatal data for creating categories and covariants for later analyses.

Another common problem in longitudinal birth cohorts is related to repeated psychometric measures. For example it is impossible to use the same psychological/neuropsychological tests for all age groups. Tests of intelligence for pre-school children, school aged children and adults are different and even though they can be scaled in corresponding distributions centering on the mean IQ of 100, they still are not fully comparable.

For the statistical analysis of new data, commercially available statistical analysis packages will be used. For obvious reasons, the statistical consulting facilities provided by the University of Helsinki will be extensively put to use.

## Ethical considerations

Infants in the original database were enrolled with an informed consent by a parent. All studies have been conducted in accordance with the Helsinki declaration and consent has been given at each phase of the follow-up. In 2001 the subjects gave their written consent for future follow-ups.

The ethical review was initially done at the Children's Hospital at the Helsinki University Central Hospital for the follow-up visits at 5, 9 and 16.

In November 2012 the material was handed over to Prof Laura Hokkanen, PhD., by a written agreement by Dr. Katarina Michelsson, MD, PhD. A new ethical review for the current project and the new plan as well as inclusion of a new group of researchers will be applied for from the Review Board of the Helsinki and Uusimaa hospital district during the spring of 2013 (Medical Research Act 488/1999). A new invitation letter will be sent out to all participants for consent.

Special care will be taken to respect the autonomy of research subjects, to avoid harm, and to ensure privacy and data protection of the cohort. Identifying information will be handled according to the Finnish Personal Data Act (523/1999).

If a subject is found to have a condition requiring medical attention, he or she will be referred to proper medical services by the responsible physician.

## Plans for dissemination of the study outcome

The results will be published in international peer reviewed scientific journals. Open access electronic publications will be preferred. It is expected that a project of this magnitude will gain publicity in national media.
