# Supplementary material for: The Perinatal Adverse events and Special Trends in Cognitive Trajectory (PLASTICITY) - pre-protocol for a prospective longitudinal follow-up cohort study
Source: F1000Res. 2013 Feb 14;2:50. [Version 1] doi: 10.12688/f1000research.2-50.v1 (PMC3790606; doi:10.12688/f1000research.2-50.v1)
Supplement: Peer review history

# Peer review: Referee response for version 1

**Published:** 2013-08-27

**Authors:** Erin Bigler

**Affiliations:** 1 Department of Psychology, Brigham Young University, UT, USA

With the improvements in managing adverse events that occur during the perinatal period, infants that would not have survived given medical and obstetrical care prior to the 1970’s now survive. Although cognitive and neurobehavioral sequelae are commonplace in children who experience adverse perinatal effects, almost all studies that have addressed such problems have been based on select sub-samples, including small N and anecdotal studies. Larger outcome studies often get allocated to one particular type of adverse perinatal even like asphyxia. The uniqueness the study design presented by Hokkanen, Launes and Michelsson is to include all “adverse” perinatal events and prospectively follow this cohort throughout life. In this study from a single maternity hospital, 1196 subjects met inclusion criteria for perinatal adverse events from a consecutive sample of 22, 359. In that these subjects were enrolled from 1971 through 1974 means they are now reaching their 40’s. The other by-product of improved healthcare of the 20th and 21st centuries is that once born, longevity is now the expectation with survival well beyond 78 years of age the norm. Because of this and the potential for those who suffer perinatal adverse events to have increased risk for a host of neurological, neuropsychiatric, cognitive and behavioral effects, it has become very important to better understand how these perinatal influences affect long-term outcome. Much of the past research has merely focused on the transition from surviving the adverse event and its influence on childhood and then the transition to adulthood. This proposed study is truly a lifespan study with a single cohort that experienced adverse perinatal events.

In this well thought-out and written study design, Hokkanen and colleagues capitalize on a single cohort of births in Finland where prospectively acquired medical, neurological, developmental, educational and neuropsychological test information has been previously obtained and the current proposed design will capture this cohort in their 40’s. All fields of medicine and psychology have improved in major ways since the 1970s with improved assessment methods and a more comprehensive understanding of how early brain insult may affect function and how it should be measured. As outlined in Figure 1, Hokkanen *et al*. show the potential interactive nature of numerous relations between neurodevelopmental disorders and later-in-life neurodegenerative disorders. Previously not assumed to be a factor in aging and the development of degenerative diseases like Alzheimer’s, early events are now know to participate in the overall cognitive and brain reserve of the individual. Certain factors may predispose to degeneration yet other factors may relate to plasticity and resiliency. Potentially, the only way some of these factors could be teased out is through long-term prospective studies as proposed by Hokkanen *et al*. Inexorably this cohort will march on to their 60’s and beyond and tracking their progress may provide immeasurable insights into what are critical vulnerability factors that predispose to mild cognitive impairment and transition to dementia. Hokkanen *et al*. nicely detail the major factors for data extraction as well as attrition. Table 3 outlines the specific outcome areas that will be targeted and methods to examine outcome. One of the critical improvements since this cohort was first identified is the development in neuroimaging. As outlined in Table 3, MRI including functional MRI studies will be obtained.

This manuscript is very well written. The questions raised, the uniqueness of this cohort, the ability to answer not only some long-term developmental questions in terms of outcome, but also aging and risk for dementia merely point out how incredibly unique is this cohort. I very much like the acronym – PLASTICITY. This has been and will be a most important and valuable clinical research cohort to study and will help answer questions about vulnerability as well as resiliency in brain development and aging.

I have read this submission. I believe that I have an appropriate level of expertise to confirm that it is of an acceptable scientific standard.

# Peer review: Referee response for version 1

**Published:** 2013-03-08

**Authors:** Chiadi Onyike

**Affiliations:** 1 Frontotemporal Dementia and Young-Onset Dementias Clinic, The Johns Hopkins Hospital, Baltimore, MD, USA

The Perinatal Adverse events and Special Trends in Cognitive Trajectory (PLASTICITY) - pre-protocol for a prospective longitudinal follow-up cohort study is rather intriguing. By observing a well-developed cohort of individuals who suffered perinatal adversity as they enter mid-life and later-life, this study can provide valuable insights into the long-term effects of perinatal adversity on the cognitive, behavioural and functional prospects of the individual. The findings would also be valuable from a neuropsychiatry research perspective, as it offers examination of perinatal and psychosocial risk factors of mid-life and late-life dementia, cognitive decline and psychiatric disorder. Already, some data suggest that learning disabilities are associated with a higher risk for frontotemporal dementia in later life (see Rogalski et al., 2008). Thus this is an excellent and timely study, and the research agenda offers examination of many important questions. As with all major undertakings, the devil is in the details. One would eventually want to see the details, i.e.., the measurement and data analysis procedures. I am especially interested in understanding how the data analysis will manage intervening events such as adverse rearing environment, head trauma, substance abuse, that may have potential for independent or overriding influence on the outcomes.

I have read this submission. I believe that I have an appropriate level of expertise to confirm that it is of an acceptable scientific standard.
